# Cognitive function in early-phase schizophrenia-spectrum disorder: IQ subtypes, brain volume and immune markers

**DOI:** 10.1017/S0033291721004815

**Published:** 2023-05

**Authors:** Andrew J. Watson, Annalisa Giordano, John Suckling, Thomas R. E. Barnes, Nusrat Husain, Peter B. Jones, Carl R. Krynicki, Stephen M. Lawrie, Shôn Lewis, Naghmeh Nikkheslat, Carmine M. Pariante, Rachel Upthegrove, Bill Deakin, Paola Dazzan, Eileen M. Joyce

**Affiliations:** 1 The Department of Clinical and Motor Neuroscience, UCL Queen Square Institute of Neurology, London, UK; 2 Department of Psychological Medicine, Institute of Psychiatry, Psychology and Neuroscience, King's College London, London, UK; 3 Brain Mapping Unit, Department of Psychiatry, Herchel Smith Building for Brain and Mind Sciences, University of Cambridge, Cambridge, UK; 4 Division of Psychiatry, Imperial College London, London, UK; 5 Division of Neuroscience and Experimental Psychology, University of Manchester, Manchester, UK; 6 MAHSC, The University of Manchester, Manchester, UK; 7 Lancashire & South Cumbria NHS Foundation Trust, Accrington, UK; 8 Cambridgeshire & Peterborough NHS Foundation Trust, Cambridge, UK; 9 Institute for Mental Health, University of Birmingham, Birmingham, UK; 10 Division of Psychiatry, Centre for Clinical Brain Sciences, University of Edinburgh, Edinburgh, UK; 11 Stress, Psychiatry and Immunology Lab & Perinatal Psychiatry, The Maurice Wohl Clinical Neuroscience Institute, King's College London, London, UK; 12 Forward thinking Birmingham, Birmingham Women's and Children's Hospital NHS Foundation Trust, Birmingham, UK; 13 National Institute for Health Research Biomedical Research Centre at South London and Maudsley NHS Foundation Trust, London, UK

**Keywords:** Cognition, inflammation, neuroimaging, psychosis, schizophrenia-spectrum disorder, subtypes

## Abstract

**Background:**

Evidence suggests that cognitive subtypes exist in schizophrenia that may reflect different neurobiological trajectories. We aimed to identify whether IQ-derived cognitive subtypes are present in early-phase schizophrenia-spectrum disorder and examine their relationship with brain structure and markers of neuroinflammation.

**Method:**

161 patients with recent-onset schizophrenia spectrum disorder (<5 years) were recruited. Estimated premorbid and current IQ were calculated using the Wechsler Test of Adult Reading and a 4-subtest WAIS-III. Cognitive subtypes were identified with k-means clustering. Freesurfer was used to analyse 3.0 T MRI. Blood samples were analysed for hs-CRP, IL-1RA, IL-6 and TNF-*α*.

**Results:**

Three subtypes were identified indicating preserved (PIQ), deteriorated (DIQ) and compromised (CIQ) IQ. Absolute total brain volume was significantly smaller in CIQ compared to PIQ and DIQ, and intracranial volume was smaller in CIQ than PIQ (*F*_(2, 124)_ = 6.407, *p* = 0.002) indicative of premorbid smaller brain size in the CIQ group. CIQ had higher levels of hs-CRP than PIQ (*F*_(2, 131)_ = 5.01, *p* = 0.008). PIQ showed differentially impaired processing speed and verbal learning compared to IQ-matched healthy controls.

**Conclusions:**

The findings add validity of a neurodevelopmental subtype of schizophrenia identified by comparing estimated premorbid and current IQ and characterised by smaller premorbid brain volume and higher measures of low-grade inflammation (CRP).

## Introduction

Cognitive dysfunction is common in schizophrenia (Meier et al., 2014; Reichenberg & Harvey, 2007) with the variation in social, functional and occupational outcomes being significantly attributable to cognitive heterogeneity (Green, 2006; Green, Kern, Braff, & Mintz, 2000). Cognitive subtypes have been identified based on differences between premorbid and post-illness onset measures of intellectual function. Most studies describe three cognitive trajectories: below average premorbid and post-onset cognition indicating long-standing impairment (compromised); at least average premorbid cognition but cognitive scores after illness onset that suggest deterioration (deteriorated); and at least average premorbid and post-onset cognition indicating preserved cognition (preserved) (Ammari et al., 2014; Badcock, Dragović, Waters, & Jablensky, 2005; Czepielewski, Wang, Gama, & Barch, 2017; Dickinson et al., 2020; Kremen, Seidman, Faraone, & Tsuang, 2008; Leeson et al., 2011; Mercado, Johannesen, & Bell, 2011; Weickert et al., 2000; Wells et al., 2015; Woodward & Heckers, 2015). We previously showed that these cognitive subtypes are present at psychosis onset in two first-episode schizophrenia-spectrum cohorts suggesting that when cognitive deterioration is present, it occurs early in the illness (Joyce, Hutton, Mutsatsa, & Barnes, 2005; Leeson et al., 2011).

There are several outstanding questions regarding the validity of these cognitive subtypes. One is whether they reflect different neurobiological trajectories (Weickert et al., 2000). Woodward and Heckers (2015) observed smaller MRI intra-cranial volumes (ICV) in a compromised subtype compared with healthy volunteers. As ICV is a proxy for premorbid brain volume, it was hypothesised that this is a neurodevelopmental subtype of schizophrenia with ‘cerebral hypoplasia’. They distinguished this from a deteriorated cognitive subgroup which showed normal ICV but smaller total brain volume (TBV) which is indicative of neurodegeneration. Supporting these findings, a different study found that neurodevelopmental and neurodegenerative MRI profiles mapped onto their conjugate cognitive subtypes (Czepielewski et al., 2017). However, Van Rheenen et al. (2018) did not find evidence for a neurodevelopmental subtype with reduced ICV. Instead, they found reduced TBVs indicative of brain shrinkage across all subtypes which was regionally more pronounced in the compromised group. This was suggestive of a continuum of progressive brain neurodegeneration across all subgroups in association with the degree of cognitive impairment. Another neurobiological mechanism which may differentiate cognitive subgroups concerns the effect of innate inflammatory responses. Several studies have shown that elevated C-reactive protein (CRP), a marker of systemic low-grade inflammation, is associated with cognitive impairment in adults with schizophrenia (Bulzacka et al., 2016; Dickerson, Stallings, Origoni, Boronow, & Yolken, 2007; Johnsen et al., 2016; Misiak et al., 2018). Other inflammatory markers, IL-6, IL-1 receptor antagonist (IL1RA), and tumour necrosis factor-alpha (TNF-a) have also been implicated but less consistently so (Frydecka et al., 2015; Misiak et al., 2018). Elevated childhood IL-6 levels have been shown to increase the risk of psychotic symptoms in young adults (Khandaker, Pearson, Zammit, Lewis, & Jones, 2014) and CRP levels to be associated with subclinical psychotic symptoms in adolescents (Khandaker et al., 2021). Furthermore, a higher erythrocyte sedimentation rate (ESR), another inflammatory marker, was associated with lower premorbid IQ and higher risk for schizophrenia in army recruits (Kappelmann et al., 2019). These findings provide tentative evidence for the hypothesis of Miller and Goldsmith (2019) that genetic and environmental factors encountered during development lead to peripheral and central inflammation which influences premorbid cognitive function and risk of schizophrenia.

A second question regarding the cognitive subtypes is whether patients with the ‘preserved’ subtype have intact cognitive function (Carruthers, Van Rheenen, Gurvich, Sumner, & Rossell, 2019). This is important because it challenges the commonly held notion that cognitive impairment is a core attribute of schizophrenia. For example, one study comparing high IQ patients and closely matched controls found no statistical difference between the two groups on a range of neuropsychological measures suggesting that cognitive function may not always be affected by the neuropathological process thought to underlie schizophrenia (MacCabe et al., 2012). However another study of schizophrenia patients and healthy controls matched for IQ, found that subtest index scores differed so that patients had worse processing speed task performance, but better verbal comprehension and perceptual organisation, a pattern present even in a high IQ subgroup (Wilk et al., 2005). This question is also relevant for the interpretation of MRI studies in the context of cognitive subtypes. For example, both Woodward and Heckers (2015) and Van Rheenen et al. (2018) found that their preserved cognitive subgroups showed a degree of brain shrinkage which suggested to them that this group might not be neuropsychologically intact.

We aimed to address these questions by applying a data-driven approach to classify the three IQ trajectory-based subtypes externally validated in previous studies (Dickinson et al., 2020); Weickert et al., 2000), to cognitive data from a new cohort of patients who had recently developed a schizophrenia-spectrum disorder. In each of the three subtypes, we measured TBV, grey matter volumes and cortical thickness and the inflammatory markers previously associated with cognition in schizophrenia: CRP, Il-6, IL1RA and TNF alpha. We also included a healthy control group to examine further the notion of intact cognition pertaining to the preserved subtype. We hypothesised that a compromised group would have smaller ICV than preserved and deteriorated groups. In comparison with a preserved group, the deteriorated group was expected to show smaller TBV after adjusting for ICV. Examining differential inflammatory marker profiles between groups was exploratory.

## Methods

To address the research questions, we performed a secondary analysis of the BeneMin clinical trial data (Deakin et al., 2018). BeneMin was designed to test whether the anti-inflammatory drug minocycline improves psychotic symptoms and cognition in patients with early-phase schizophrenia spectrum disorder, recruited within 5 years of onset. CRP, proinflammatory cytokines and MRI brain volume were measured as potential biomarkers of effect. We restricted the analysis to baseline data before allocation of patients to the investigational drug or placebo.

### Participants

Schizophrenia spectrum disorder: Patients were recruited from 11 UK mental health trusts as part of a double-blind randomised-controlled trial (BeneMIn) assessing the potential benefit of minocycline on negative symptoms in early-phase psychosis (Deakin et al., 2018). Ethical approval was obtained from the North West Research Ethics Committee (ref.11/NW/0218). All participants met the criteria for schizophrenia spectrum disorder using the Mini-International Neuropsychiatric Interview (MINI) and were aged 16-35 years at the onset of psychosis, currently receiving care from UK NHS Early Intervention Services (EIS) and within the first 5 years of their first presentation to mental health services. All had at least mild persisting psychotic symptoms, defined by a score >2 on the delusions, hallucinations, suspiciousness or conceptual disorganisation items on the Positive and Negative Syndrome Scale (PANSS) (Kay, Fiszbein, & Opler, 1987). Exclusion criteria were alcohol or substance abuse seriously affecting function; inability to communicate fluently in English; estimated WTAR premorbid IQ of <70; and current suicide or violence risk. Participants were required to be taking stable antipsychotic treatment from a mental health care team (Lisiecka et al., 2015). Psychotic symptoms were assessed with the PANSS (Kay et al., 1987). Depression was assessed with The Calgary Depression Scale for Schizophrenia (CDSS) (Addington, Addington, & Schissel, 1990) and social function with the self-report Social Function Scale (SFS) (Birchwood, Smith, Cochrane, Wetton, & Copestake, 1990) and observer-rated Global Assessment of Functioning (GAF) scale. Lifetime cannabis use was assessed using a self-report measure and current BMI was calculated as both can effect inflammatory measures (Mastinu et al., 2018; Visser, Bouter, McQuillan, Wener, & Harris, 1999).

Out of 207 participants in the BeneMin trial, 166 were included in this study based on the completion of cognitive tests. All measures and the MRI scans were performed before allocation to placebo or minocycline conditions (Deakin et al., 2018).

To examine the question of intact cognition in the preserved subtype, we used equivalent age-matched neuropsychological data from 82 healthy volunteers who took part in the West London First-Episode Psychosis Study and were recruited by advertising in local job centres, schools and hospitals. Exclusion criteria were a personal history of psychiatric illness or a history of such illness in any first-degree relatives, previous head injury, a neurological or endocrine disorder known to affect brain function, and drug or alcohol abuse (Leeson, Barnes, Hutton, Ron, and Joyce, 2009)

Of those with complete cognitive data, 141 completed MRI scans, nine of these were excluded due to motion artefacts and two due to data acquisition errors, leaving data for 130 scans. Inflammatory markers were available for 138 participants with complete cognitive assessments after exclusions. Five did not complete a blood-draw and those with hsCRP > 10 were excluded (*n* = 10), as this is thought to reflect the presence of an acute infection rather than systemic inflammation (Osimo, Baxter, Lewis, Jones, & Khandaker, 2019). The remaining exclusions were due to outliers (*n* = 13).

### Neuropsychological assessments

Premorbid IQ was estimated using the Wechsler Test of Adult Reading (WTAR) which is co-normed against the WAIS-III. Current IQ was estimated with a short form of the WAIS-III (Blyler, Gold, Iannone, & Buchanan, 2000), developed for use in schizophrenia. This uses 4 subtests: Digit Symbol, Information, Block Design and Arithmetic for the calculation of pro-rated full-scale IQ. The Rey Auditory Verbal Learning Test (AVLT) (Lezak, Howieson, Loring, Hannay, & Fischer, 2004) was used to assess verbal learning and memory in which participants were read a list of 15 nouns and asked to recall as many as possible immediately afterwards on each of five trials.

### Neuroimaging

3 T MRI scans were performed at each of the study sites. The MRI sequences were coordinated across imaging centres by author JS based on the previous NeuroPsygGrid multi-centre validation and reliability study (Suckling et al., 2012) comprising three-dimensional T1-weighted magnetisation-prepared rapid gradient-echo (MPRAGE/SPGR) as described in Deakin et al. (2018). Whole-brain segmentation and cortical reconstruction were carried out by author AG using FreeSurfer v5.3.0 (Massachusetts General Hospital, Harvard Medical School; http://surfer.nmr.mgh.harvard.edu). The fully automated procedure used has been described by Fischl et al. (2002). All volumes and thickness measures were visually inspected after the segmentation pipeline and no manual edits were necessary. In contrast to the ‘cortex’ measure, ‘total grey volume’ also includes subcortical and cerebellum volumes. Participant sex and age, and MRI acquisition centre were included as covariates in between-group analyses.

### Measures of inflammation

Blood samples were collected in a 9 ml ethylenediamine tetraacetic acid tube and spun in a centrifuge, within four hours of collection, for 15 min at 20 degrees Celsius and 2000 g. Measurements were of high-sensitivity C-reactive protein (hs-CRP), Interleukin 1-receptor antagonist (IL-1RA), Interleukin 6 (IL-6), and Tumour Necrosis Factor-alpha (TNF-*α*). Details of the assays can be located in the online Supplementary materials.

### Analyses

The three cognitive subgroups were classified with data-driven cluster analysis according to the method of Weickert et al. (2000). As the WTAR is a reading test it can be inaccurate for estimating IQ in dyslexia or when English is not the first language, we excluded participants with a WTAR IQ of more than 10 IQ points below WAIS IQ for both the patient group (*n* = 5) and healthy controls (*n* = 35). Estimated premorbid IQ (WTAR score) and current WAIS IQ score were entered into a non-hierarchical iterative *k*-means cluster analysis with the number of clusters set to ‘3’, to create three clusters with the greatest separation after allowing for iterations.

We used linear discriminant function analysis (DFA) on the three-cluster solution to ascertain the relative accuracy of the IQ variables in predicting cluster membership. Homogeneity of variance was confirmed using Box's *M* test (*p* = 0.124). To assess the reliability of the classification model, a ‘leave-one-out’ classification was performed.

The resulting clusters were analysed using one-way analysis of variance (ANOVA) or analysis of covariance (ANCOVA) for normally distributed continuous cognitive measures. χ^2^ was used for demographic and categorical data. Logarithm transformations were used on non-normally distributed continuous data. *Z* score transformations of patient scaled scores were calculated in relation to the mean and standard deviation of healthy control scaled scores on each of the IQ subtests.

Due to differences between the healthy volunteer and patient groups, sex was entered as a covariate in all cognitive analyses comparing patients with healthy controls. For the imaging analysis, sex, age and scan site were controlled for when comparing groups. BMI and lifetime cannabis use were controlled for as potential confounders in the analysis of inflammation markers (Mastinu et al., 2018; Visser et al., 1999).

Raw *p* values are reported for ANOVAs. To control for multiple testing, main effects were adjusted for false discovery rate (FDR) using the Benjamini–Hochberg method (Benjamini & Hochberg, 1995). Cognitive, clinical, functioning, imaging and inflammation variables were tested separately. For main effects significant after this adjustment (*p* = <0.05), post-hoc between-group comparisons were made using Bonferonni corrections (Bland & Altman, 1995). Cohen's effect sizes (ES) were calculated (Cohen, 1998).

## Results

### Demographics (Table 1)

There was no significant difference in age between the patient and healthy control groups [*t* (242) = 3.72, *p* = 0.06]. There were more males in the patient group [χ^2^ (1) = 18.19, *p* = <0.001] and the control group had more years of education [*t* (242) = −3.99, *p* = <0.001]. The patient sample had moderate symptoms and difficulty in social functioning, as indicated by mean PANSS total [67.62 (14.48)] and GAF [55.81 (10.65)] scores (see online Supplementary Table S1). There were no differences between cognitive subtype groups in symptom scores and social function (see online Supplementary Table S2).

**Table 1.** Demographics of patients, controls, and empirically derived cognitive clusters

	Patients (*n* = 161)	Controls (*n* = 82)			Comparison	
	Mean (s.d.)	Mean (s.d.)			Main Effect (χ^2^/F)	
Age	25.36 (5.02)	26.83 (6.61)			*t* (242) = 3.72, *p* = 0.06	
Sex (% Male)	77.01%	50.00%			χ^2^ (1) = 18.19, *p* = **<0.001**	
Years of Education	12.80 (1.89)	14.20 (2.03)			t (242) = −3.99, *p* = **<0.001**	

	HC (*n* = 82) Mean (s.d.)	PIQ (*n* = 57) Mean (s.d.)	DIQ (*n* = 61) Mean (s.d.)	CIQ (*n* = 43) Mean (s.d.)		*post-hoc*^a^
Age	26.83 (6.61)	25.63 (5.64)	24.30 (4.48)	26.28 (4.51)	*F*_(__3242)_ = 2.50, *p* = 0.06	
Sex (% Male)	50.00%	82.45%	70.49%	76.74%	χ^2^ (3) = 19.18, *p* = **<0.001**	HC < PIQ, DIQ, CIQ
Years of Education	14.20 (2.03)	13.20 (1.93)	12.92 (2.06)	11.72 (1.12)	*F*_(__3242)_ = 8.58, *p* = **<0.001**	PIQ **>** CIQ; HC **>** DIQ + CIQ
Lifetime Cannabis Use (% Yes)	*	72.2%	47.5%	65.1%	χ^2^ (2) = 0.59, *p* = 0.745	

HC, Healthy Controls; PIQ, Preserved IQ; DIQ, Deteriorated IQ; CIQ, Compromised IQ.

a following Bonferroni correction.

### Cognition (Table 2)

**Fig. 1.**
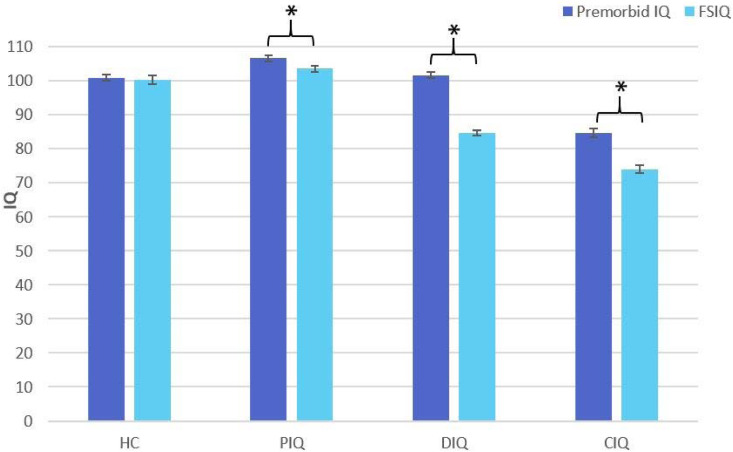
Bar chart comparing estimated premorbid IQ and full-scale current IQ scores for cognitive subtypes and healthy controls. HC, healthy controls; PIQ, preserved IQ group; DIQ, deteriorated IQ group; CIQ, compromised IQ group; FSIQ, pro-rated Full-scale IQ. * denotes statistically significance. Error bars represent standard error of mean (s.e.).

K-means cluster analysis with groups set to ‘3’ showed good cluster stability with few iterations. DFA (χ^2^ (4) = 102.85, *p* < 0.001, canonical correlation = 0.921): showed high correct classification rates (96.4%). with the overall classification accuracy remaining high in the leave-one-out analysis (94.5%) (See online Supplementary Table S3). 35% of patients were classified as putatively preserved (PIQ), 38% as deteriorated (DIQ) and 27% as compromised (CIQ). The estimated premorbid IQ of the healthy controls (HC), PIQ and DIQ groups was in the average range whereas that of the CIQ group was in the low average range. Figure 1 shows that the estimated premorbid IQ of the PIQ group was higher than the HC group and that PIQ and HC estimated current IQ was equivalent, with a small but significant fall in IQ in the PIQ group [*t* (56) = 2.99, *p* = 0.004]; the DIQ group had a significant fall in mean IQ into the low average range [*t* (60) = 12.49, *p* < 0.001]; and the CIQ group also showed significant deterioration into the below-average range [*t* (42) = 6.95. *p* < 0.001].

**Table 2.** Comparison of group cognitive function

	HC (*n* = 82)	PIQ (*n* = 57)	DIQ (*n* = 61)	CIQ (*n* = 43)	Comparison							
	Mean (s.d.)	Mean (s.d.)	Mean (s.d.)	Mean (s.d.)	Main Effect (F)	*post-hoc*^a^	Effect Size (d)					
							HC v. PIQ	HC v. DIQ	HC v. CIQ	PIQ v. DIQ	PIC v. CIQ	DIQ v. CIQ
Premorbid IQ^a^	100.96 (8.78)	106.75 (6.91)	101.75 (6.80)	84.74 (7.93)	*F*_(__3, 239)_ = 71.31, *p* **< 0.001**	PIQ **>** HC, DIQ **>** CIQ	0.7*	0.1	1.9**	0.7*	3.0**	2.3**
FSIQ^a^	100.34 (11.70)	103.59 (6.67)	84.72 (6.80)	74.04 (7.95)	*F*_(__3, 239)_ = 127.22, *p* **< 0.001**	HC, PIQ **>** DIQ **>** CIQ	0.3	1.8**	2.6**	2.8**	4.0**	1.4**
Digit Symbol^a^	9.24 (2.27)	7.53 (2.79)	5.93 (1.94)	4.93 (1.56)	*F*_(__3, 239)_ = 44.83, *p* **< 0.001**	HC **>** PIQ > DIQ, CIQ	0.7*	1.6**	2.2**	0.7*	1.2**	0.6*
Block Design^a^	10.29 (2.52)	11.45 (2.39)	8.22 (2.29)	7.02 (2.09)	*F*_(__3, 239)_ = 37.52, *p* **< 0.001**	PIQ > HC **>** DIQ, CIQ	0.5*	0.8**	1.4**	1.3**	1.9**	0.5*
Information^a^	11.39 (2.45)	12.49 (2.29)	9.22 (2.57)	6.88 (2.03)	*F*_(__3, 239)_ = 55.43, *p* **< 0.001**	PIQ **>** HC **>** DIQ **>** CIQ	0.5*	0.9**	2.0**	1.4**	2.6**	1.1**
Arithmetic^a^	9.41 (2.81)	10.73 (2.05)	7.44 (2.33)	5.60 (2.31)	*F*_(__3, 239)_ = 43.60, *p* **< 0.001**	PIQ > HC **>** DIQ > CIQ	0.5*	0.8**	1.5**	1.5**	2.3**	0.8**
AVLT Total^a^	49.86 (8.82)	42.76 (11.19)	37.25 (8.71)	32.04 (10.28)	*F*_(__3, 236)_ = 37.95, *p* **< 0.001**	HC **>** PIQ **>** DIQ **>** CIQ	0.7*	1.4**	1.9**	0.5*	1.0**	0.5*

a Sex was entered as a covariate for comparisons with healthy controls. *indicates medium effect size, ** indicates large effect size. HC, healthy controls; PIQ, preserved IQ group; DIQ, deteriorated IQ group; CIQ, compromised IQ group; FSIQ, full-scale IQ; AVLT, Auditory Verbal Learning Test. Bold font denotes significance following FDR correction. Scaled scores reported.

In general, the PIQ group performed better than the DIQ group on all domains who in turn performed better or equivalent to the CIQ group. When compared with HCs, the PIQ group performed significantly better on block design, information and arithmetic tests subtests but significantly worse on the digit symbol test [ES 0.7]. Verbal learning was also significantly worse [ES 0.7] in PIQ than HCs (Table 2).

**Fig. 2.**
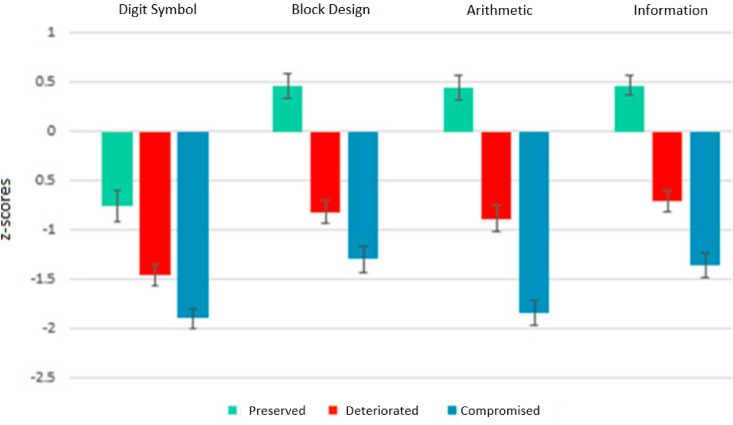
IQ cognitive sub-domain *z* scores relative to healthy control performance. PIQ, preserved IQ group; DIQ, deteriorated IQ group; CIQ, compromised IQ group. Error bars represent the standard error of the mean (s.e.).

Figure 2 shows the standardised z-scores of the patient groups for each IQ subtest in relation to HC performance; 95% CIs for HCs – Digit Symbol: [8.74–9.74]; Arithmetic: [11.88–13.09]; Block Design: [9.73–10.84]; Information: [10.85–11.93]. In general, the PIQ group performed better than the DIQ group on all domains who in turn performed better or equivalent to the CIQ group. When compared with HCs, the PIQ group performed significantly better on block design, information and arithmetic tests subtests but significantly worse on the digit symbol test [ES 0.7]. Verbal learning was also significantly worse [ES 0.7] in PIQ than HCs (Table 2).

### Neuroimaging (Table 3)

Intracranial volume (ICV) was significantly larger in the PIQ group than the CIQ group. Absolute total brain volume (aTBV) was significantly smaller in the CIQ group than both the DIQ and PIQ groups. When controlling for ICV, differences were not found between groups on measures of TBV, cortical volume, or grey matter volume. There was no difference between groups on a measure of mean cortical thickness.

**Table 3.** Between-group comparison of grey matter volume and thickness (cm^3^)

	PIQ (*n* = 48)	DIQ (*n* = 47)	CIQ (*n* = 35)	Comparison Effect Size (d)				
	Mean (s.e.)	Mean (s.e.)	Mean (s.e.)	Main Effect (F)	*post-hoc*^1^	PIQ v. DIQ	PIQ v. CIQ	DIQ v. CIQ
Total Intracranial Volume^2^	1508.77 (17.46)	1473.65 (17.80)	1411.31 (20.31)	*F*_(__2, 124)_ = 6.407, *p* **= 0.002**	PIQ > CIQ	0.2	0.8**	0.4
Absolute Total Brain Volume^2^	1137.80 (14.80)	1115.38 (15.06)	1052.024 (17.22)	*F*_(__2, 124)_ = 7.453, *p* **= 0.001**	PIQ + DIQ > CIQ	0.2	0.8**	0.5*
Total Brain Volume^3^	1109.82 (7.35)	1112.18 (7.34)	1094.67 (8.65)	*F*_(__2, 123)_ = 1.302, *p* = 0.276				
Cortex^3^	460.96 (4.92)	455.52 (4.91)	446.69 (5.79)	*F*_(__2, 123)_ = 1.697, *p* = 0.188				
Total Grey Volume^3^	622.63 (5.38)	617.26 (5.37)	606.33 (6.33)	*F*_(__2, 123)_ = 1.871, *p* = 0.158				
Average Cortical Thickness^2^	2.41 (0.2)	2.39 (0.02)	2.36 (0.02)	*F*_(__2, 124)_ = 2.123, *p* = 0.124				

PIQ, Preserved IQ; DIQ, Deteriorated IQ; CIQ, Compromised IQ.

*indicates medium effect size, ** indicates large effect size.

1 After Bonferonni correction.

2 Controlling for age, sex and site.

3 Controlling for ICV, age, sex and site.

### Inflammatory markers (Tables 3 and 4)

Between-group comparisons were performed on log10 transformed measures, adjusting for age, sex, BMI and cannabis use. There was a significant difference in hsCRP levels. Post-hoc tests showed that the PIQ group had significantly lower hsCRP levels than the CIQ patients, which remained after correction for multiple comparisons. There were no between-group differences on any of the other inflammation measures.

**Table 4.** Between-group comparison of log-transformed values for hsCRP and cytokines

	PIQ (*n* = 49)	DIQ (*n* = 54)	CIQ (*n* = 35)	Comparison		Effect Size (d)		
	Mean (s.d.)	Mean (s.d.)	Mean (s.d.)	Main Effect (F)	*post-hoc*	PIQ v. DIQ	PIQ v. CIQ	DIQ v. CIQ
hs-CRP	0.00 (0.55)	0.16 (0.62)	0.33 (0.47)	*F*_(__2, 131)_ = 5.01, *p* = **0.008**	PIQ **<** CIQ	0.3	0.6*	0.4
IL-1RA^1^	2.49 (0.29)	2.48 (0.30)	2.52 (0.25)	*F*_(__2, 131)_ = 0.18, *p* = 0.829	–			
IL-6^1^	−0.21 (0.29)	−0.26 (0.25)	−0.16 (0.19)	*F*_(__2, 132)_ = 0.71, *p* = 0.493				
TNF-a^1^	0.36 (0.09)	0.39 (0.10)	0.38 (0.11)	*F*_(__2, 131)_ = 2.29, *p* = 0.172				

PIQ, Preserved IQ: DIQ, Deteriorated IQ: CIQ, Compromised IQ.

*indicates medium effect size

1 Controlling for age, sex, BMI and cannabis smoking status. Mean raw hsCRP scores [s.d.]: PIQ group = 1.94 [2.18]; DIQ group = 2.84 [2.70]; CIQ group = 3.18 [2.36].

## Discussion

In this study, we replicated our previous findings of three cognitive subtypes in early-phase schizophrenia-spectrum disorder using a data-driven approach in a new cohort of patients (Joyce et al., 2005). These corresponded to preserved (PIQ, normal estimated premorbid and current IQ), deteriorated (DIQ: normal estimated premorbid and low current IQ) and compromised (CIQ: low premorbid and current IQ) subgroups (Weickert et al., 2000). We examined the relationship between cognitive subtype and MRI-derived ICV, brain volume and cortical thickness to assess whether these subtypes reflect differences in neurobiological trajectories (Weickert et al., 2000). The compromised subgroup had a significantly smaller aTBV than both the preserved and deteriorated subgroups, indicative of a smaller brain. The compromised group also had a smaller ICV compared with the preserved group. When ICV was entered as a covariate in the analysis, the difference in aTBV in the compromised group was no longer evident. As ICV is a proxy for premorbid brain volume, this suggests that smaller brain size in the compromised group was neurodevelopmental. Intracranial and brain volume increase in parallel during childhood. From early adolescence, ICV is static but brain volume begins to decrease as a reflection of normal brain maturation. Abnormally reduced brain volume is thought to reflect neurodegenerative processes whereas reduced ICV indicates an early neurodevelopmental deficit (Woodward & Heckers, 2015). Therefore our finding of smaller brain size in the compromised group may reflect premorbid intellectual function linked to early brain development. This finding supports that of previous studies comparing cognitive subgroups with healthy controls showing reduced ICV only in the compromised subgroups (Czepielewski et al., 2017; Woodward & Heckers, 2015). These studies also found evidence for neurodegeneration in patients with a deteriorated cognitive trajectory. Compared to controls, TBV was reduced but ICV was normal suggesting that brain shrinkage had occurred after maximal brain growth. We were unable to find evidence of this in relation to cognitive decline in the deteriorated subgroup, which would have been evident with a smaller brain volume adjusted for ICV compared with the preserved subgroup (Woodward & Heckers, 2015). Possible explanations are that neurodegeneration is present in all subtypes but we did not have a healthy control group to ascertain this (Van Rheenen et al., 2018; Woodward & Heckers, 2015); that our patients were earlier in the illness course and progressive atrophy was not yet evident (Van Rheenen et al., 2018); or that we did not have the statistical power to detect differences between the PIQ and DIQ subgroups. Van Rheenen et al. (2018), in a large study which included healthy controls, did not find the reduced total ICV in a compromised subgroup and instead found reduced brain volume, corrected for ICV, in all subgroups. The compromised subgroup had more pronounced reductions in global and specific volumetric measures and also uniquely smaller volumes in certain frontal and temporal cortical areas. Thus, this study demonstrated a continuum of brain neurodegeneration in association with cognitive function and evidence of specific, earlier brain changes indicative of an additional neurodevelopmental process in those with evidence of premorbid compromised intellectual function.

Taken together, these findings support the validity of defining compromised cognitive subgroups using low estimated premorbid and current cognitive function as indicative of abnormal brain development (Van Rheenen et al., 2018). Whether defining a specific deteriorated cognitive subgroup is in itself biologically meaningful requires further study, as both Woodward and Heckers (2015) and Van Rheenen et al. (2018) found evidence of MRI brain shrinkage in their preserved cognitive subgroups when compared to healthy controls. This suggests that there is a continuum of neurodegeneration across all cognitive subgroups. To support the notion that the extent of MRI identified neurodegeneration is associated with the degree of impaired cognition, they suggested that ‘preserved’ subgroups are not neuropsychologically intact. In our study, although there was no significant difference between the estimated current IQ of the PIQ group and healthy controls, the PIQ group showed a small but significant decline from their own estimated premorbid IQ. The PIQ group also showed an aberrant IQ subtest profile compared to healthy controls, with worse performance on processing speed (digit symbol subtest) and better performance on information, block design and arithmetic subtests. This group was also worse than controls on the auditory verbal learning and memory task. This accords with the findings of Wilk et al. (2005) who compared WAIS index scores in schizophrenia patients and healthy controls, closely matched for IQ, and found patients were worse on processing speed but better on verbal comprehension and perceptual organisation. Other studies have also found that those with seemingly intact cognition can be separated from healthy controls by poorer performance on tests of processing speed (González-Blanch et al., 2011; Heinrichs et al., 2015) and verbal memory (Hill, Beers, Kmiec, Keshavan, & Sweeney, 2004). These findings add weight to the evidence that impaired processing speed and verbal memory are evident in patients even when matched with healthy controls for IQ, suggesting that cognitive impairment is a fundamental feature of schizophrenia (Gray, McMahon, & Gold, 2013; Wilk et al., 2005). They also support the proposal of Carruthers et al. (2019) that ‘preserved’ subgroups be relabelled as ‘relatively intact’ when classified according to the cognitive trajectory methodology in future studies.

When we examined markers of inflammation in the cognitive subtypes we found that all subgroups had moderate (>1.0–<3.0 mg/l) to high (>3.0 mg/l) CRP levels (Metcalf et al., 2017) with a linear relationship, so that the PIQ group had the lowest level and the CIQ group the highest. The CIQ group had a significantly higher mean CRP level than the PIQ group even after controlling for confounders. These findings support an association between higher low-grade inflammation and worse cognitive performance rather than inflammation being specific to a particular cognitive subgroup. In support of this, several studies have found a relationship between current cognitive function (including IQ) and CRP in schizophrenia (Bulzacka et al., 2016; Dickerson et al., 2007; Johnsen et al., 2016; Misiak et al., 2018). Kappelman et al., (2019), in a large epidemiological study, found a linear association between lower premorbid IQ and higher ESR at the time of recruitment to the Swedish army. They also showed that lower premorbid IQ was a robust risk factor for a subsequent diagnosis of schizophrenia and that premorbid IQ partly mediated the ESR-psychosis relationship. They concluded that low-grade inflammation is a factor influencing cognitive development and schizophrenia risk. In a meta-analysis of population-based longitudinal studies, Osimo et al. (2019) found an association between high CRP at baseline (>3.0 mg/l) and the subsequent development of schizophrenia thus supporting low-grade inflammation being a risk factor for this disorder. Although we found the highest CRP in the group with the lowest estimated premorbid IQ, this was cross-sectional and it therefore cannot be concluded that high CRP was present before psychosis onset and related to compromised IQ. Future longitudinal developmental studies are required to investigate this possibility

Significant group differences in inflammatory markers were limited to CRP only. CRP serum concentration is known to be elevated in response to inflammation, though evidence suggests that CRP is not only a marker, but also important in the mediation of inflammatory processes and expression of cytokines (Sproston & Ashworth, 2018). The mechanisms underlying this induction and exact relationships with the expression of individual cytokines remain unclear. One possible explanation for group differences in CRP, is that its transcription is induced by a combination of essential and supplementary inflammatory responses resulting from the overall acute phase response (Weinhold, Bader, Poli, & Rüther, 1997; Zhang, Sun, Samols, & Kushner, 1996). Longitudinal studies are needed to investigate whether this inflammation is chronic, and the mechanisms leading to its induction.

We did not find significant differences between groups on measures of clinical symptoms or global and specific social functioning. Lack of differences in clinical symptoms is perhaps unsurprising, given that severity symptoms and cognition have largely been found to be independent (Heaton et al., 2001), though some studies in long-standing schizophrenia have shown evidence of more severe negative symptoms in compromised groups (Czepielewski et al., 2017; Van Rheenen et al., 2018). More surprising is the lack of differences in functional outcomes, given that cognition has been shown to be the best predictor of outcome following the onset of psychosis. Kendler Ohlsson, Mezuk, Sundquist, & Sundquist (2016) found that deviation from IQ level of biological relatives, rather than IQ score itself, confers the greatest risk for schizophrenia and it may be this differential which impacts differences in functional outcomes. Another possible explanation is that functional differences are not adequately captured by the measures used in this study.

### Limitations

This study has several limitations. The main one is that, because this was a secondary analysis, we were constrained by data collected in the original study (Deakin et al., 2018). All our patients were taking antipsychotic medication, but we did not have sufficient detail to assess medication effects. Furthermore, whilst we endeavoured to control for potential confounds of inflammatory status, we did not have data to adjust for smoking quantity or other health-related variables which could influence inflammation. We also did not have a healthy control group recruited at the same time and were unable to infer whether brain volumes or inflammation differed relative to controls. Furthermore, whilst the WTAR has been shown to be a reliable hold measure and is cross-validated with the WAIS-III, It would undeniably be preferable to have the same measure of IQ pre- and post-illness onset in a prospective cohort study. Finally, despite a relatively good overall sample size, stratifying participants into subgroups resulted in reduced statistical power. Caution in the interpretation of the results is therefore necessary, particularly with regard to the brain and inflammation markers.

## Conclusion

Taken together our findings add validity to the presence of a neurodevelopmental subtype of schizophrenia (Weickert et al., 2000; Woodward & Heckers, 2015), characterised in this study by smaller premorbid TBVs and higher measures of low-grade inflammation. However, we were unable to confirm that those with a deterioration in IQ from premorbid estimates had MRI evidence of brain shrinkage. Our finding of high CRP in the compromised group may indicate that low-grade inflammation contributes to abnormal brain development and premorbid cognitive function. Finally, we found that even when patients are thought to have preserved cognitive function, based on premorbid and current estimates of IQ, impaired verbal memory and processing speed are evident. This supports the notion that cognitive impairment is a fundamental feature of schizophrenia and which needs to be taken into account when investigating cognitive subtypes (Carruthers et al., 2019).

## Supplementary material

For supplementary material accompanying this paper visit https://doi.org/10.1017/S0033291721004815. click here to view supplementary material
