# Supplementary material for: Cognitive function in early-phase schizophrenia-spectrum disorder: IQ subtypes, brain volume and immune markers
Source: Psychol Med. 2022 Feb 18;53(7):2842–51. doi: 10.1017/S0033291721004815 (PMC10244009; doi:10.1017/S0033291721004815)
Supplement: Supplementary file 1 [file S0033291721004815sup001.docx]

**Supplementary Materials**

***Measures of Inflammation***

A venous blood sample was collected at baseline using 9ml VACUETTE® EDTA tubes for the extraction of six aliquots of 0.5 ml of plasma. The aliquots were stored at -80^o^C in each individual site, where the collection took place, and three of the aliquots were subsequently transferred at King’s College London for the measurement of the hs-CRP, IL-1RA, IL-6, and TNF-α, whereas the other three aliquots were kept stored in each site as backup.

The samples were centrifuged and the plasma supernatant was extracted and stored at -80^o^C within four hours of withdrawal. Bloods for plasma CRP marker was measured using the Cormay anti-CRP antibody (PZ Cormay SA, Lomianki, Poland) sensitised to latex particles and for IL-1RA ELISA kit supplied by R&D Systems was used, which employs the quantitative sandwich enzyme immunoassay technique. Plasma cytokines were measured using Meso Scale Discovery (MSD) sandwich immunoassays. V-PLEX Plus Proinflammatory Panel 1 Human Kit was used to detect the levels of IL-6 and TNF-α levels, and plates read on an MSD QuickPlex SQ 120, as previously conducted (Nettis et al., 2020; Russell et al., 2019) . The inter-assay coefficient of variations was <10%. The results were analysed using MSD DISCOVERY WORKBENCH analysis software. Assays were performed at King's College London.

Nettis, M. A., Veronese, M., Nikkheslat, N., Mariani, N., Lombardo, G., Sforzini, L., … Pariante, C. M. (2020). PET imaging shows no changes in TSPO brain density after IFN-α immune challenge in healthy human volunteers. *Translational Psychiatry*, *10*(1), 89. https://doi.org/10.1038/s41398-020-0768-z

Russell, A., Hepgul, N., Nikkheslat, N., Borsini, A., Zajkowska, Z., Moll, N., … Pariante, C. M. (2019). Persistent fatigue induced by interferon-alpha: A novel, inflammation-based, proxy model of chronic fatigue syndrome. *Psychoneuroendocrinology*, *100*, 276–285. https://doi.org/10.1016/j.psyneuen.2018.11.032

**Supplementary Table 1 (S1): Clinical and social functioning of entire patient sample**

|  | **Patient Group** |
| --- | --- |
|  | **Mean (SD)** |
| PANSS Positive Symptoms | 16.55 (4.86) |
| PANSS Negative Symptoms | 17.31 (5.73) |
| PANSS General Symptoms | 33.77 (7.62) |
| PANSS Total Symptoms | 67.62 (14.48) |
| Calgary Depression Scale |  |
|  |  |
|  |  |
| SFS Social Withdrawal | 103.53 (13.84) |
| SFS Relationships | 114.25 (17.45) |
| SFS Independence Performance | 104.17 (12,90) |
| SFS Recreation | 106.25 (17.00) |
| SFS Prosocial | 107.83 (16.10) |
| SFS Independence Competence | 109.37 (12.30) |
| SFS Employment | 104.36 (10.87) |
| SFS Total | 107.09 (9.74) |
| GAF | 55.81 (10.65) |
|  |  |

SFS = Social Functioning Scale, GAF – Global assessment of Functioning

**Supplementary Table 2 (S2): Between-group comparison of symptoms and social functioning**

PIQ, Preserved IQ: DIQ, Deteriorated IQ: CIQ, Compromised IQ,  ^a^Does not survive FDR correction

|  | **PIQ (n = 55)** | **DIQ (n = 61)** | **CIQ (n = 43)** |  |
| --- | --- | --- | --- | --- |
|  | **Mean (SD)** | **Mean (SD)** | **Mean (SD)** | **Main Effect (F)** |
|  |  |  |  |  |
| Positive Symptoms | 16.42 (4.87) | 16.74 (4.70) | 16.44 (5.18) | F (2, 156) = 0.07, p = 0.928 |
| Negative Symptoms | 17.11 (5.90) | 16.95 (6.08) | 18.07 (6.25) | F (2, 156) = 0.52, p = 0.592 |
| General Symptoms | 33.16 (6.96) | 33.75 (8.51) | 24.58 (7.19) | F (2, 156) = 0.66, p = 0.662 |
| Total Symptoms | 66.69 (13.19) | 67.44 (15.89) | 69.09 (14.17) | F (2,156) = 0.41, p = 0.714 |
| Calgary Depression | 5.65 (4.69) | 5.04 (4.14) | 4.27 (4.52) | F (2, 156) = 2.85, p = 0.240 |
|  |  |  |  |  |
|  |  |  |  |  |
| Social Withdrawal | 105.09 (12.57) | 104.18 (14.66) | 100.83 (14.53) | F (2, 156) = 1.06, p = 0.348 |
| Relationships | 117.05 (17.19) | 114.16 (18.91) | 110.02 (14.61) | F (2, 156) = 1.38, p = 0.254 |
| Independence Performance | 105.16 (13.01) | 103.12 (12.96) | 105.17 (12.91) | F (2, 156) = 0.47, p = 0.621 |
| Recreation | 108.27 (18.72) | 105.88 (16.06) | 104.84 (16.02) | F (2, 156) = 0.63, p = 0.533 |
| Prosocial | 110.08 (14.03) | 104.55 (16.87) | 109.64 (16.50) | F (2, 156) = 2.79, p = 0.064 |
| Independence Competence | 111.21 (12.40) | 109.10 (11.30) | 107.05 (13.35) | F (2, 155) = 1.17, p = 0.312 |
| Employment | 107.56 (11.22) | 102.79 (11.01) | 102.92 (9.71) | F (2, 156) = 3.86, p = 0.023^a^ |
| SFS Total | 109.20 (9.88) | 105.95 (9.74) | 105.92 (9.31) | F (2, 155) = 2.05, p = 0.132 |
| GAF | 56.98 (10.52) | 55.46 (12.61) | 54.77 (7.32) | F (2, 157) = 0.57, p = 0.564 |
|  |  |  |  |  |

**Supplementary Table (S3) Results of the Discriminant Function Analysis for Cluster Solution**

| **Group** | **DFA results** | **Classification model type** | **Cluster (%)** | | | |
| --- | --- | --- | --- | --- | --- | --- |
| **Patient** | Function 1: (*χ* ^2^ (4) = 102.85, p <.001, canonical correlation =.921):  Highest loading: Current IQ (r=.81) | Group |  | PIQ | CIQ | DIQ |
|  |  | Original | PIQ | 100 | 0 | 0 |
|  |  |  | CIQ | 0 | 87.5 | 14.3 |
|  |  |  | DIQ | 0 | 0 | 100 |
|  |  |  |  |  |  |  |
|  |  | Cross-validated | PIQ | 100 | 0 | 0 |
|  |  |  | CIQ | 0 | 85.7 | 14.3 |
|  |  |  | DIQ | 0 | 0 | 100 |
|  |  | 96.4% of original grouped cases  94.5% of cross-validated grouped cases correctly classified | | | | |

**Supplementary Table 4 (S4): Between group comparison of BMI**

|  | **PIQ (n=57)**  Mean (SD) | **DIQ (n=61)**  Mean (SD) | **CIQ (n=43)**  Mean (SD) | **Main Effect** |
| --- | --- | --- | --- | --- |
| Body Mass Index (BMI) | 26.29 | 27.88 | 28.88 | F (2,152) = .23, P = .787 |

PIQ, Preserved IQ: DIQ, Deteriorated IQ: CIQ, Compromised IQ
